# Autochthonous Leishmaniasis in the United States of America

**DOI:** 10.3390/microorganisms13112485

**Published:** 2025-10-30

**Authors:** Chaoqun Yao, Yi Yang, Aifang Du

**Affiliations:** 1 Department of Biomedical Sciences, Ross University School of Veterinary Medicine, Basseterre P.O. Box 334, Saint Kitts and Nevis; 2 One Health Center for Zoonoses and Tropical Infectious Diseases, Ross University School of Veterinary Medicine, Basseterre P.O. Box 334, Saint Kitts and Nevis; 3 Institute of Preventive Veterinary Medicine, College of Animal Sciences, Zhejiang University, Hangzhou 310058, China; yangyi0607@zju.edu.cn (Y.Y.); afdu@zju.edu.cn (A.D.); 4 Zhejiang Provincial Key Laboratory of Preventive Veterinary Medicine, College of Animal Sciences, Zhejiang University, Hangzhou 310058, China

**Keywords:** autochthonous leishmaniasis, *Leishmania*, USA, transmission, sand fly

## Abstract

Leishmaniasis is endemic in 99 countries worldwide, including the USA where it has low endemicity. The disease is emerging but likely underdiagnosed due to its historical absence in the diagnostic differentials of American physicians. Additionally, the public seems to have little knowledge about this disease. Here, a comprehensive literature review was carried out on autochthonous leishmaniases in humans in the USA, including their associated *Leishmania* spp., capable sand fly vector, transmission route, and risk to the parasitic infection. All 89 cases were cutaneous leishmaniasis reported in Texas, Oklahoma, Arizola, and North Dakota. The collective information should serve to mitigate both autochthonous and imported leishmaniasis by expanding reservoir and vector surveillance and improving physician training in diagnosis in the USA.

## 1. Introduction

Leishmaniasis is an emerging/re-emerging parasitic disease caused by over 20 *Leishmania* spp. protozoa. It clinically manifests in three major forms, i.e., cutaneous leishmaniasis (CL), visceral leishmaniasis (VL), and mucocutaneous leishmaniasis [1,2]. Among them, CL is the most benign, often displaying chronic skin lesions on the frequently exposed body parts such as the head and extremities. This form is known to last several months to over a year, followed by self-healing even without proper treatment. In contrast, VL is the most severe form of the disease, usually resulting in the death of more than 90% of patients if left untreated [3,4,5]. Annually, there are approximately 0.6−1.0 million new CL cases and 50−90 thousand VL cases worldwide across 99 endemic countries according to the World Health Organization (WHO, https://www.who.int/news-room/fact-sheets/detail/leishmaniasis, accessed on 24 October 2025). The USA is among the 99 endemic countries and has a few species of wild mammals serving as reservoirs including *Neotoma micropus* (Southern Plains woodrats), *N. floridana* (eastern woodrat), *N. albigula* (White-throated woodrat), and *Peromyscus attwateri* (Texas deermice) [6,7]. Leishmaniasis has been a notifiable disease since 2007 in Texas (https://www.dshs.texas.gov/2007-annual-report, accessed on 24 October 2025).

The rareness of leishmaniasis in the USA makes a timely diagnosis very challenging. Autochthonous leishmaniases in humans have been reported infrequently. In addition, U.S. residents, just like those in non-endemic countries, may acquire infections internationally when they travel to highly endemic areas such as South America, the Indian subcontinent, and Mediterranean regions [8,9,10]. Furthermore, considering the effects of climate change, there is a trend for the disease to spread to more northern latitudes [11]. Consequently, there needs to be a pivotal effort to draw medical doctors’ attention to include this disease in their clinical differential list as well as to educate general populations. Here, we comprehensively review autochthonous leishmaniasis in humans, in aspects of its distribution within the USA, routes of transmission, various parasite species, sand fly vector, and risk to infection.

## 2. Case Identification

### 2.1. Identification of Leishmania spp.

The gold standard for the diagnosis of leishmaniasis is the microscopic finding of amastigotes within macrophages in biopsied tissues/organs/aspirates. Positive in vitro culture of promastigotes from these biological materials is also considered a confirmed diagnosis no matter whether a positive microscopic finding is obtained. Molecular techniques for positive identification of *Leishmania* spp. are preferred, as the morphologic similarity of both amastigotes and promastigotes makes discrimination between species difficult, even by electron microscopy (EM). Isoenzyme profile analysis, PCR, and DNA sequencing are useful techniques to identify *Leishmania* spp. PCR and DNA sequencing have become increasingly popular since the 2000s and have almost completely replaced isoenzyme profiling that was widely used since the 1980s. Lately, metagenomic next-generation sequencing has been applied in diagnosing various pathogens including *Leishmania* spp. [12,13].

### 2.2. Criteria for Autochthonous Leishmaniasis

A confirmed case of human leishmaniasis described in the literature must meet at least one of three criteria to have been considered USA-autochthonous, i.e., locally acquired in the USA, and thus included in the current study: (1) a confirmed case of patients who did not, in the course of their lifetime, travel outside of the USA; (2) a confirmed case of patients who did not travel to an endemic foreign country in the immediate 5 years prior to leishmaniasis onset; (3) a confirmed case of a military personnel or veteran who had not been deployed to Iraq, Kuwait, or Afghanistan since Operation Desert Storm in 1990.

## 3. Autochthonous Leishmaniasis

**Figure 1:**
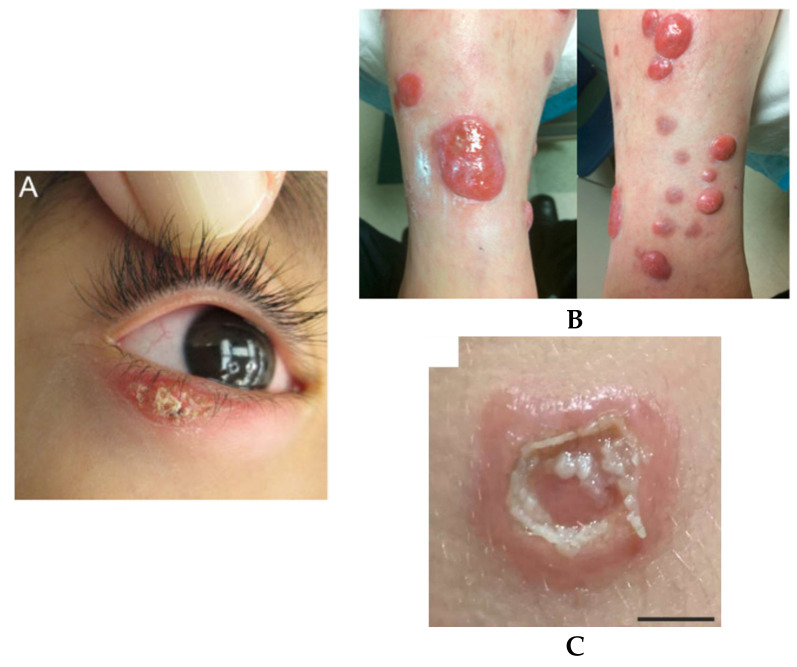
Typical and atypical gross lesions of autochthonous cutaneous leishmaniasis in the U.S. population caused by *Leishmania donovani* complex (**A**) and *L. mexicana* (**B**,**C**). (**A**) From [24] with permission for reuse. (**B**) From [25] with permission for reuse; the largest lesion ~4.0 × 4.5 cm. (**C**) From [28] with permission for reuse; the left arm; scale bar: 1 cm.

Until now, all autochthonous leishmaniases recorded in humans in the USA are CL. A typical CL case clinically manifests as an ulcerated skin lesion that is often located on exposed body parts such as the head and extremities (Figure 1). The first undisputable autochthonous leishmaniasis that meets the criteria outlined in the Section 2 was reported by Stewart and Pilcher in 1945. The patient was a six-year-old boy who presented to a doctor on 10 October 1942, with ulcerated skin lesions on the dorsum of both feet, right knee, and right buttock. A biopsy of these lesions revealed amastigotes microscopically, which confirmed the diagnosis of leishmaniasis. The boy was born in Alice, Texas and had never traveled out of Texas prior to the onset of the disease [10]. Since then, 89 autochthonous cases in total have been reported (Table 1). The patients originated from four different U.S. states. The states and case numbers are Texas—84, Oklahoma—2, Arizona—2, and North Dakota—1.

**Table 1:** Autochthonous human leishmaniasis in USA (open cells indicate no data available).

Year	State	Age	Sex	Clinical Signs and Symptoms	Diagnostic Methods *	*Leishmania* spp.	Treatment (/F/H/LTF/NR) ^‡^	References
1942	TX	6	M	Chronic ulcers on the dorsum of both feet, right knee, and right buttock	M			[10]
1967	TX	64	F	Numerous nodules and plaques over extremities and buttocks for years	C, M		Camolar (F)	[14]
1972	TX	74	F	Hard plaque on right eyelid, left cheek, and left earlobe	AI, M		Surgery (H)	[15] ^€^
1974	TX	56	M	Crusting and superficial ulceration of the left cartilaginous septum	AI, C, M, S, VI		Pentostam (F)	
1980	TX	11	M	Red papule on the left cheek	C, IP, M	*L. mexicana*	Sodium stibogluconate (H)	[16,17]
1982	TX	56	F	Crusted plaque on left earlobe	M, S		Antimony potassium tartrate (H)	
1982	TX	5	M	Papule on left front thigh	M, S		Surgery (H)	
1983	TX	10	M	Papules on face	C, M, S		No treatment (H)	
1986	TX	46	F	Nodules on right arm and wrist	M, C, IP	*L*. *mexicana*	Ketoconazole (H)	[18]
1986	TX	13	M	Ulcer on right ear	M		Antimony tartrate (H)	[19]
1986	TX	28	F	Ulcerating papule on right cheek	M		Unknown (LTF)	
1988	TX	52	M	Crusted lesion on right ear	M		Isoniazid (H)	
1988	TX	37	F	Ulcerating nodule on right lower leg	M		No treatment (H)	
1988	TX	3	M	Papule on nose	M, C		Cryotheropy (LTF)	
1988	TX	4	M	Nodule on left lower eyelid	M, C		No treatment (H)	
1988	TX	50	F	Ulcerating papule on nose	M		No treatment (H)	
1988	TX	2	M	Plaque on left cheek	M, C		No treatment (LTF)	
1989	TX	28	F	Ulcerating nodule on right cheek	M, C		Ketoconazole (H)	
1989	TX	20	F	Ulcerating nodule on right angle	M		Antimony tartrate (H)	
1989	TX	86	F	Ulcer on right cheek	M		Ketoconazole (H)	
1989	TX	2	M	Ulcer on right ear	M		Antimony tartrate (H)	
1989	TX	62	M	Nodule on right ear, right cheek, forehead and right elbow; plaque on right thigh	M, C		Sodium stibogluconate (F); Sodium stibogluconate + Ketoconazole (H)	
1989	TX	67	M	Ulcer on left ear	M		No treatment (H)	
1990	TX	81	F	Ulcerating papule on right cheek	M		Electrodesiccation + curettage (H)	
1992	TX	15	M	Lesion on face	M		Isoniazid + Rifampin (H)	
1993	TX	51	F	Ulcerating nodule on left wrist	M, C		Ketoconazole (H)	
1987	TX	62	M	Cutaneous lesions on the extremities	M, C			[20]
2002 ^+^	TX	78	F	Erythematous plaque on right forearm	M		No treatment (LTF)	[21]
2003	OK	26	M	Skin lesion on right cheek	M		No treatment (H)	[22]
2005	OK	73	M	Skin lesions on right forearm	M		Heat (LTF)	
2005	TX	74	F	Nodule on left eyelid	M		No treatment (LTF)	
2005	TX	70	M	Skin lesion on right arm	M		Amphotericin + Fluconazole (NR)	[11,22] ^#^
2005	TX	8	F	Skin lesions on face and left upper arm	M, C, PCR ^#^	*L. mexicana*	Amphotericin B + Fluconazole (NR)	
2006	TX	60	F	Skin lesion on nose	M		Ketoconazole + Cryotherapy (NR)	
2006	TX	76	F	Skin lesion on forehead	M		Cryotherapy (NR)	
2006	TX	80	M	Skin lesion on left arm	M			
2006	TX	64	M	Skin lesion on right abdomen	M			
2007	TX	57	M	Skin lesion on left back	M		Diflucan + Surgery (NR)	
2007	TX	82	F	Skin lesion on left cheek	M		Fluconazole (NR)	
2007	TX	64	F	Skin lesion on right chest	M			
2006	TX		M	Skin lesion on right abdomen	M			[23]
2007	TX		F	Skin lesion on upper arm	M, PCR	*L. mexicana*		
2007	TX		F	Skin lesions on chin and neck	M			
2007	TX		F	Skin lesions on forearm and wrist	M, PCR	*L. mexicana*		
2010	TX		M	Skin lesion on left wrist	M			
2011	TX		F	Skin lesions on face, left elbow, and buttock	M, PCR	*L. mexicana*		
2011	TX		F	Skin lesion on upper arm	M			
2012	TX		M	Skin lesion on face	M, PCR	*L. mexicana*		
2013	TX		F	Skin lesion on right wrist	M, PCR	*L. mexicana*		
2013	TX		F	Skin lesion on upper arm	M			
2013	TX		F	Skin lesion on right forehead	M, PCR	*L. mexicana*		
2013	TX		F	Skin lesion on forehead	M			
2013	TX		M	Skin lesion on forearm	M			
2013	TX		F	Skin lesion on upper arm	M, PCR	*L. mexicana*		
2013	TX		F	Skin lesion on shoulder	M			
2013	TX		M	Skin lesion on left arm	M			
2013	TX		F	Skin lesion on right lower eyelid	M			
2013	TX		F	Skin lesion on left eyelid	M			
2013	TX		F	Skin lesion on upper shoulder	M, PCR	*L. mexicana*		
2014	TX		M	Skin lesion on upper eyelid	M, PCR	*L. mexicana*		
2014	TX		F	Skin lesion on face	M, PCR	*L. mexicana*		
2014	TX		F	Skin lesion on left temple	M, PCR	*L. mexicana*		
2014	TX		M	Skin lesions on arm	M, PCR	*L. mexicana*		
2014	TX		F	Skin lesions on face and eyelid	M			
2014	TX		F	Skin lesion on right forehead	M			
2014	TX		F	Skin lesion on left cheek	M, PCR	*L. mexicana*		
2014	TX		M	Skin lesion on right ear	M, PCR	*L. mexicana*		
2014	TX		F	Skin lesion on left upper arm	M			
2015	TX		F	Skin lesion on left upper arm	M			
2015	TX		M	Skin lesions on face and cheek	M, PCR	*L. mexicana*		
2015	TX		F	Skin lesion on right dorsal hand	M			
2015	TX		F	Skin lesion on forearm	M, PCR	*L. mexicana*		
2015	TX		F	Skin lesion on left earlobe	M			
2015	TX		M	Skin lesions on elbows	M			
2015	TX		M	Skin lesion on left forearm	M			
2015	TX		F	Skin lesion on face	M			
2015	TX		M	Skin lesions on ear and back	M, PCR	*L. mexicana*		
2016	TX		F	Skin lesion on right anterior upper neck	M			
2016	TX		M	Skin lesion on right upper arm	M, PCR	*L. mexicana*		
2016	TX		F	Skin lesion on left forehead	M, PCR	*L. mexicana*		
2016	TX		F	Skin lesion on right face	M			
2012	ND	2	M	Single lesion each on the upper and lower eyelid of right eye	PCR	*L. donovani* complex	No treatment (H)	[24]
2016	TX	67	M	Multiple painless and non-pruritic papules at the anterior surface of the right leg	M, C, PCR, D	*L. mexicana*	Miltefosine + Ketoconazole (F)	[25]
2017	AZ	72	F	Two discrete, edematous, violaceous papules on the low back	M, C, PCR, D	*L. ellisi*	No treatment (H)	[26,27]
2018–2019	TX	2	F	Non-healing nodular lesion on right jawline	^¥^	*L. mexicana*	Fluconazole (H)	[28]
	TX	3	M	Non-healing nodular lesion on left arm	M, PCR, D	*L. mexicana*	Fluconazole (H)	
	TX	0 ^£^	M	Nodular lesion on face	M, PCR, D	*L. mexicana*	Fluconazole (F); Paromomycin (F)	
2020 ^+^	TX	65	M	Three erythematous lesions on the left lateral shoulder	M		Cryotherapy (H)	[29]
2023 ^+^	AZ	34	M	Single ulcerated verruous plague on lower left leg	M, I		Surgery (H)	[30]

*: AI: animal infection; C: culture; D: DNA sequencing; I: immunohistochemistry; IP: isoenzyme profile; M: microscopy of amastigotes; PCR: polymerase chain reaction; S: serology; VI: sand fly vector infection; ^‡^: F: failure; H: healed; LTF: lost to follow; NR: not reported; ^€^: also included an additional asymptomatic case of 16-year-old female identified by serology in 1975 [31]; ^+^: publication year; ^#^: reported by both [22] and [11]; ^¥^: genetic analysis by the Centers for Disease Control and Prevention (CDC); ^£^: 6 months old.

Among eighty-nine cases, only twenty-eight (31.5%) have *Leishmania* spp. firmly identified by molecular techniques, i.e., PCR and/or DNA sequencing (twenty-six cases) and isoenzyme profiling (two cases). Specifically, all 26 cases in Texas are infected by *L*. *mexicana*; the remaining two from Arizona and North Dakota are infected with *L*. *ellisi* and *L*. *donovani* complex, respectively (Table 1). *Leishmania donovani* complex consists of *L. donovani* and *L. infantum*, which usually causes VL in endemic areas in the Indian subcontinent, Brazil, and the Mediterranean area, respectively. Nevertheless, infection with both species also results in CL [32,33,34]. Therefore, the CL case due to infection of *L*. *donovani* complex in North Dakota is not extraordinary.

**Figure 2:**
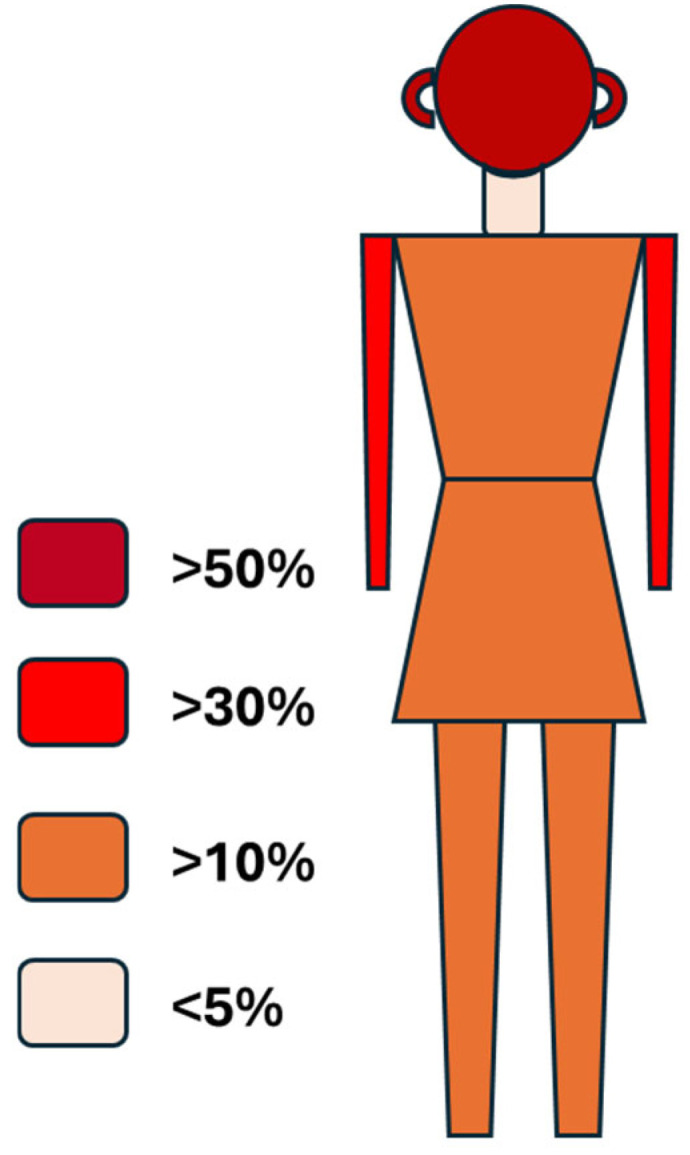
Distribution of autochthonous cutaneous leishmaniasis in the USA on human body parts. The body parts are categorized into head, neck, trunk, upper limp, and lower limp. Percentage of the body parts affected is presented as shown in the legend from the highest (>50%) on the head to the lowest (<5%) on the neck.

Sex, age and lesional location on body parts of all reported cases are tabulated in Table 1. Forty (44.9%, n = 89) were male and forty-nine (55.1%) were female. There is no statistical difference between this ratio of 40:49 and the presumed equal ratio of 44.5:44.5 (χ^2^ = 0.4562, *p* = 0.50), which suggests both sexes are equally susceptible to the disease without much gender bias. These are similar to the epidemiological data of CL in Iran [35,36], but are different from the epidemiological data of VL in Brazil, which have demonstrated that VL is more common in males than in females after puberty [37,38]. In the latter, such male susceptibility to symptomatic VL has been experimentally confirmed to be biological in murine models [38]. As far as onset age, only 48 cases have recorded ages of patients ranging from infant (few months old) to 86 years old. Specifically, the ages are <10 years–11, ≥10–5, ≥25–18, and ≥65–14. Two thirds (32/48) are 25 years or older, which suggests a likelihood of professional/adventure exposure, although this needs to be confirmed. Regarding the location of skin lesions on various body parts, the head (including ears and nose) is the most common, accounting for 53.9% (48/89), followed by the upper limb 32.6% (29/89), lower limb 11.2% (10/89), and trunk 10.1% (9/89), with the least on the neck at 2.2% (2/89) (Figure 2). The total number of body parts affected are more than 89 (the total number of patients) as a few patients had more than one body part affected. These data clearly show that often exposed body parts (such as the head and the upper limb) are more likely to sustain sand fly bites, hence having the greatest chance of being infected by the *Leishmania* parasites and leading to leishmaniasis. Interestingly, the neck, another often exposed body part, is the least of the lesional locations. The reason why the neck is much less attractive to sand fly bites is unknown at this time. Further studies are warranted and may provide informational clues to develop preventions to minimize sand fly bites on the head and upper limb where almost 90% of the bites occur.

Since 94.4% (84/89) of the confirmed autochthonous cases are in Texas (Table 1), it is worthy of further discussion. An autochthonous case of CL was firmly diagnosed by EM in a 78-year-old woman with a 4-month history of a skin lesion on her right forearm in Washington County, Texas. An additional 29 cases were collected, which were all from south-central Texas [21]. Nevertheless, nine CL cases were later found at the Dallas-Fort Worth metroplex in 2005–2007 [11,22]. The latest cases involving three children occurred between October 2018 and April 2019. Their ages were 2 years, 3 years, and six months old. Two lived in Ellis County and were only 9 miles from one another; the third in Grayson County [28]. These latest cases show an unequivocal trend of the northward spread of the disease in the state, which is a clearly alarming movement which should trigger a response by medical and public health professionals.

In 2007, leishmaniasis became a notifiable disease in Texas. The annually reported cases are as follows: 2007—nine; 2008—zero; 2009—two; 2010—zero; 2011—four; 2012—six; 2013—eleven; 2014—twelve; 2015—six; 2016—thirteen; 2017—eight; 2018—fifteen; 2019—ten; 2020—not available; 2021—nine; 2022—eleven; 2023—ten; and 2024—four (https://www.dshs.state.tx.us/IDCU/data/annual/Texas-Annual-Reports-2000s.aspx, accessed on 24 October 2025). Nevertheless, determining whether all of these were autochthonous cases is very challenging. In 2018, McIlwee BE and colleagues investigated the increasing number of cases in Texas. They used laboratory information systems to find each patient’s travel history for all patients reported from 2007 to 2017. Forty-one out of sixty-nine reported cases (59%) had no history of travel outside the USA. Consequently, they were all considered autochthonous (Table 1) [23].

The case that originated in North Dakota is interesting and worthy of further discussion. First, out of all 89 autochthonous cases, it is the only case that was infected with *L. donovani* complex (*L. donovani* and *L. infantum*) [24] that usually causes VL in humans (although rare, CL cases without a VL history have been reported) [39]. Second, among the four tests performed, only PCR was positive for parasite DNA of *L. donovani* complex [24], whereas the remaining three were negative. The latter included microscopic findings, parasite cell culture performed by the Center for Disease Control and Prevention (CDC), and antibodies to K-39 antigen using the immunochromatographic strip test. Third, the boy’s family (the parents and older brother) had just immigrated to the USA from Nepal in 2009, which is the year before the boy was born in the USA in March 2010 [24]. It is plausible that the mother was pregnant with the boy prior to immigration to the USA and he acquired the parasite through vertical transmission. Nepal is an endemic country with VL and had recorded over 1000 annual cases before 2009, and 935 cases in 2009 when the family emigrated from the country [40]. A pertinent question to ask is whether the boy was congenitally infected, considering the timing of his birth in relation to family immigration history. Unfortunately, no data on testing the boy’s mother for leishmaniasis are presented in the original publication. There are several reports of such transmission, including cases occurring in non-endemic areas, which will be further discussed later in the section on transmission routes. Fourth, the authors of the reported case speculated that case was transmitted by sand flies, and possibly had a canine origin since leishmaniasis is endemic in Foxhound populations in many U.S. states (although possibly not in North Dakota). It might even be possible that there is an anthroponotic origin of the parasite as the authors also suggested the likelihood of the boy’s parents transporting the infected vector from Nepal to North Dakota [24].

## 4. *Leishmania* spp. in the USA

**Figure 3:**
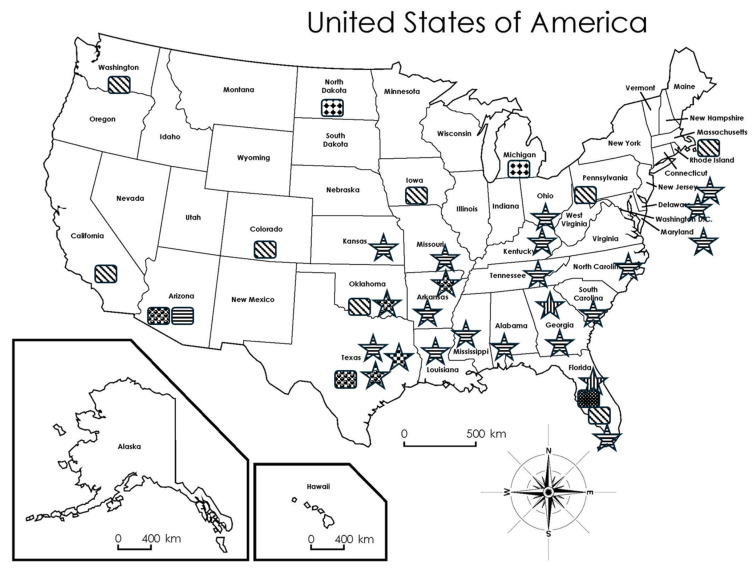
Risk levels of individual states for leishmaniasis endemicity in the USA by the distribution of *Leishmania* parasites and capable sand fly vectors with solid confirmation. *Leishmania* spp. data are from Table 1 and references [41,42,43]. Sand fly vectors *Lutzomyia* spp. are from reference [7,44]. *L. mexianan* in Arizona was identified in white-throated woodrat (*Neotoma albigula)* [45]. *Leishmania* spp: *L. mexicana*: 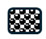; *L. infantum*: 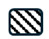; *L. donovani*: 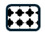; *L. ellisi*: 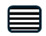; *L. martiniquensis*: 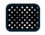; Sand fly species: *Lu. anthophora*: 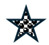; *Lu. cruciate*: 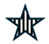; *Lu. diabolica*: 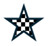; *Lu. shannoni*: 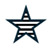. Super Teacher Worksheets—www.superteacherworksheets.com.

As described earlier in the section of case identification, identification of *Leishmania* spp. almost exclusively depends upon molecular techniques including isoenzyme profile, PCR, DNA sequencing, and metagenomic next-generation sequencing. In all, five *Leishmania* spp. have been confirmed in the USA, accounting for approximately one fourth of all human-infecting *Leishmania* spp. worldwide. All human cases in the USA are caused by *L. mexicana* except two cases—one by *L. donovani* complex and the other by *L. ellisi* (Table 1). *Leishmania ellisi* is the newest member in the genus that causes CL (Table 1) [26]. Interestingly, parasites identified from autochthonous canine cases are exclusively *L. infantum* [41]; and the parasites infecting horses are *L. martiniquensis* [42,43], which has been found causing human CL in Martinique and Thailand (1). Geographical distribution of the five *Leishmania* spp. is presented in Figure 3.

The U.S. isolates of *L. mexicana* are worthy of a brief discussion since they appear to have a unique genotype. In an early study, 72 *L. mexicana* isolates from North and Latin America and the Caribbean were analyzed for the profile of 20 isoenzymes. The isolates included were from Texas—eight, Mexico—thirteen, Belize—six, Guatemala—twelve, Ecuador—twelve, Venezuela—five, and the Dominican Republic—eight. It was found that all eight Texan isolates were highly similar to the central American ones [46]. Recently, by means of DNA sequencing, Texan *L. mexicana* isolates of a unique genotype have been found in ITS2, i.e., A → C647 and T → C649, which is different from the Mexican and South American isolates. The genotype can be a very useful tool for identifying autochthonous *L. mexicana* infection in the USA [25,28,47].

## 5. Transmission Routes

*Leishmania* spp. are vector-borne kinetoplastid protozoa that are naturally transmitted by sand fly vectors [48,49]. Lately, they have been confirmed to be vertically transmitted from mother to offsprings [50]. In addition, they may also be horizontally passed between individuals by blood transfusion. We discuss here the roles that these various transmission routes play in the U.S. endemicity of leishmaniasis.

### 5.1. Sand Fly Vector

*Leishmania mexicana* parasites were successfully isolated from naturally infected sand flies in the USA for the first time in October 1991. In this study, promastigotes were proliferatively grown from three of the twenty-seven sand flies (*Lutzomyia anthophora)* collected in Texas; two of them were identified as *L. mexicana* by isoenzyme profiling [51]. In a separate study, sand flies were collected using a CDC light trap on the personal property of a patient with autochthonous CL in Caldwell County, Texas. Fly species were identified by morphology and PCR followed by DNA sequencing targeting the mitochondrial cytochrome c oxidase subunit I. Bloodmeal sources of engorged sand flies were determined by hemi-nested PCR targeting the 16S rRNA gene. *Leishmania* spp. infection of the sand flies was determined by PCR targeting the *Leishmania* spp. rRNA-ITS2 region, followed by DNA sequencing. The fly species identified included *Lu. shannoni* (n = 1), *Lu. texana* (n = 3), and *Lu. anthophora* (n = 188). Two of six *Lu. anthophora* engorged females with bloodmeal were found to be of human origin. More importantly, 4 out of 138 female flies were PCR positive for *Leishmania* spp. DNA, one of which was found to be identical to the *L. mexicana* DNA sequence found in the infected human on the property [25]. However, whether the sand fly infected with the human isolate of *L. mexicana* was one of two *Lu. anthophora* that contained human blood was not clear. *Lutzomyia anthophora* and *Lu. vexator* were trapped in the residence of a patient in Tarrant County, Texas [22]. *Lutzomyia anthophora*, *Lu. diabolica,* and *Lu. texana* were trapped at leishmaniasis foci in Medina County and Bexar County, TX. A total of 1 out of 347 female *Lu. anthophora* was found infected with *Leishmania* sp. promastigotes, whereas none of the 494 *Lu. diabolica* and 83 *Lu. texana* were positive [52]. Under laboratory conditions, female *Lu. longipalpis* sand flies reared in the laboratory were allowed to feed on Foxhound dogs naturally infected with *L. infantum*. They were then dissected 10 days later. Promastigotes were found in the stomodeal valve of the sand flies. Furthermore, the infected sand flies were allowed to feed on lab-reared hamsters. The blood of these hamsters was tested for leishmanial DNA using qPCR. Four of seven hamsters were positive between two and four months after sand fly blood feeding. Parasite DNA was amplified by the same qPCR in the spleen, bone marrow, and lymph nodes of most of the 15 hamsters at the end of the experiment when they were sacrificed [53]. This experiment clearly shows that *L. infantum* passed down generations among Foxhounds by congenital transmission is capable of infecting sand flies, and the infected sand flies can infect mammalian hosts. Therefore, those *L. infantum*-infected Foxhounds in the USA certainly pose some risk to humans.

Collectively, four species of *Lutzomyia* spp. sand flies in the USA are considered capable vectors of *Leishmania* parasites. These are *Lu. anthophora*, *Lu. cruciate, Lu. diabolica*, and *Lu. shannoni* (Figure 3) [7,44]. In addition, *Lu. vexator*’s role as a vector waits to be confirmed and this species has been found in the States of AL, AR, CA, CO, FL, GA, KS, LA, MD, MO, MT, NM, NY, OH, OK, TN, TX, VA, WA, WY [44].

### 5.2. Vertical Transmission—Congenital Transmission

In 1986, a male infant in Kenya was diagnosed with leishmaniasis at four months of age along with his mother. He was born a premature baby and had been in the hospital all the time prior to diagnosis. Furthermore, he had been sick since day 6 of his birth, suggesting a transplacental transmission [54]. Kenya is an endemic country of leishmaniasis. Similarly, congenital transmission also happens in non-endemic regions. An eight-month-old boy born in Ukraine to a mother who had been treated for leishmaniasis during weeks 28–32 of gestation was also confirmed suffering from the disease. The boy was delivered by cesarean section in Ukraine, which is a non-endemic country for leishmaniasis, and had not traveled outside of the country. The mother had worked in Alicante, Spain, which is a leishmaniasis-endemic region. It is very likely the boy is infected by vertical transmission [55]. A 16-month-old German boy was diagnosed with leishmaniasis by high titer antibodies to *Leishmania* sp. and positive cell culture from the liver and bone marrow. The boy had never traveled beyond Germany. His mother had been to Portugal, Malta, and Corse—all endemic areas—before giving birth to him. Furthermore, the mother had antibodies to the cultured parasites originated from the boy, suggesting that the mother had been exposed to the same parasite, and that the boy was congenitally infected with the parasite [56]. The second case of congenital transmission in Germany was a 9-month-old girl without a travel history out of the country; she was diagnosed with leishmaniasis by serology, immunoblot, microscopic detection of amastigotes in bone marrow, and molecular techniques. The latter includes PCR and restriction fragment length polymorphism with the parasite being confirmed as *L. infantum*. The girl’s mother had vacations annually in Spain—an endemic country—and was serologically positive to *L. donovani*, indicating congenital transmission [57]. The case of a two-year old boy in ND [24] may likely represent an example of vertical transmission in the USA as discussed earlier.

### 5.3. Horizontal Transmission—Blood Transfusion

Another likely route of *Leishmania* transmission is horizontal transmission through blood transfusion. In the USA, leishmaniases caused by blood transfusion have been reported only in pet dogs. Three of seven recipients of seropositive blood donors developed antibodies to *Leishmania* sp. One was further confirmed with *L. infantum* infection by microscopic demonstration of amastigotes, and positive cell culture. In contrast, none of the 25 recipients of seronegative blood donors tested positive [58]. A systematic review on the global prevalence of *Leishmania* infections among blood donors was carried out, covering databases between 1997 and 2016. Among 13,743 blood donors, the seropositive rate was 7% (95% CI: 5−8%) with the lowest and highest of 0.25% and 16% in Bangladesh and Brazil, respectively. Furthermore, a 2% (95% CI: 1−3%) positive rate was yielded by molecular testing with the lowest and highest of 0.05% and 7% in Iran and Spain, respectively [59]. A total of 500 blood samples from donors in the northeastern region of Brazil were tested for *Leishmania* DNA by PCR, and 6.2% (95% CI: 4.1−8.3%) were positive. One was further determined to be *L. infantum* by DNA sequencing, which is the parasite endemic in the region [60]. In north-western Iran, 860 blood donors were tested for *Leishmania* antibodies and DNA between 2017 and 2018. A total of 2.8% were seropositive and 45% were positive for *Leishmania* kDNA [61]. In China, metagenomic next-generation sequencing was applied to detect various microbes in 10,720 plasma samples collected from blood donors during 2012−2018; a total of 1.37% were positive for *Leishmania* spp. [62]. Collectively, blood donors worldwide may carry *Leishmania* parasites in donated blood and products, which poses some risk to recipients.

Can *Leishmania* parasites stay viable long enough to be delivered to recipients under the conditions for blood product storage? It has been found that current standard storage conditions for donated blood banks are insufficient to kill *Leishmania* spp. Live cells of *L. tropica* and *L. donovani* were found in blood products under these conditions. The blood products tested included whole blood, packed red blood cells, frozen RBC, and platelet concentrate [63]. In three endemic areas of Brazil, six out of thirteen blood recipients from *L. infantum*-positive donors were followed up for 60 days, and two converted seropositive [64]. Furthermore, hamsters that were peritoneally injected with whole blood or purified monocytes prepared from clinically asymptomatic donors but serologically positive to *Leishmania* established infections [65]. Without a doubt, these lines of evidence together confirm that blood donors of *Leishmania*-positive status, even without clinical manifestations, can transmit the parasites to blood-transfusion recipients.

## 6. Risks of *Leishmania* spp. Transmission in the USA

The USA is one of the 99 countries of endemic leishmaniasis according to WHO (https://www.who.int/news-room/fact-sheets/detail/leishmaniasis, accessed on 24 October 2025). In order to maintain endemicity in an area, two components are necessary, i.e., leishmanial parasites and capable sand fly vectors. Figure 3 provides detailed information on both of these components in individual U.S. states. Three states have both necessary factors: Texas, Oklahoma, and Florida. Consequently, the risks for leishmanial transmission are highest among these three U.S. states. Arizona is also facing almost similar levels of risks since autochthonous infections have been found in both humans and wildlife, even though current information on capable sand fly vectors is lacking. Several other states (AL, AR, DE, GA, KY, KS, LA, MD, MO, MS, NC, NJ, OH, SC, TN) have also been confirmed to have capable vectors; however, even though a lower level of risk exists, human travel and animal transporting across state borders must be considered. The sole human case with *L. donovani* complex in North Dakota apparently indicates transmission from a dog to a human [24]. Therefore, *L. infantum*-infected Foxhounds should receive appropriate and immediate veterinary treatment with proper biosecurity measures to minimize risk to people, or even be euthanized when treatment fails to minimize any risk of transmission.

## 7. Conclusion Remarks

Despite being endemic in the USA, leishmaniasis has attracted little attention from medical doctors. It is highly likely that the situation in this country will turn from bad to worse if no urgent actions take place. Several factors are exceedingly important and need to be taken into consideration to mitigate the disease and its spread. First, global warming and climate change are predicted to spread the disease northwards [66]. This trend has already been observed in Texas where the disease is now found in central and northern counties from its earlier limit to southern counties [11]. Second, wild animals are infected with and serve as reservoirs for zoonotic *Leishmania* spp. [6]. All five *Leishmania* spp. found in the USA are capable of infecting humans in addition to other mammals. Third, the disease is an emerging infectious disease and a threat to public health in the USA [7]. Capable sand fly vectors exist in various states (Figure 3), which makes the spread of the disease to humans a high probability. Fourth, U.S. populations travel more frequently than ever nationally and internationally to high endemic countries/regions, which greatly increases their risks of infections.

In order to mitigate the spread of leishmaniasis, accurate and timely diagnosis followed by prompt treatment is key. These medical actions should serve to dramatically minimize the source of infections. Reducing exposure to sand fly vectors by wearing insect repellents and covering up body parts when necessary is critical (https://www.cdc.gov/leishmaniasis/hcp/clinical-overview/index.html, accessed on 24 October 2025). Educating general populations about the disease and its prevention should also be a priority. Last but not the least, efforts to combat global warming on a national and international scale should decelerate or even stop the northbound spread of this serious vector-born parasitic disease. In conclusion, there is an urgent need to expand reservoir and vector surveillance and improve physician training in diagnosis in the USA.

## Data Availability

No new data were created or analyzed in this study. Data sharing is not applicable to this article.
